# Isolated Distal Radius Fracture Reductions in Adult Emergency Department Patients in a Large Healthcare System

**DOI:** 10.5811/westjem.48817

**Published:** 2026-02-10

**Authors:** Steven C. Mahnke, Vanessa H. Newburn, Carolina D. Hooper, Aidan F. Mullan, Fernanda Bellolio, Daniel Fiterman Molinari

**Affiliations:** Mayo Clinic, Department of Emergency Medicine, Rochester, Minnesota

## Abstract

**Introduction:**

Distal radius fractures account for up to 18% of fractures in older adults and up to 20% of all fractures treated in the emergency department (ED). These fractures often require reduction and immobilization, with different modalities to provide analgesia. Our objective in this study was to summarize the management for distal radius fracture reductions in the real world of community and academic EDs.

**Methods:**

We identified adult visits for isolated distal radius fractures over a four-year period across three academic and 18 community hospital EDs from more than 490,000 per annum total visits. Visits were grouped by whether they were reduced, or not, in the ED. Reductions were further categorized by use of ultrasound-guided nerve block (UGNB), procedural sedation, or hematoma block. We recorded patient demographics, age, sex, race and ethnicity, and Emergency Severity Index scores. Our primary outcome was patient-reported pain scores (0–10 scale) at presentation and prior to disposition. Secondary outcomes were total milligrams of morphine equivalents administered, ED length of stay and 30-day ED return rates.

**Results:**

There were 3,642 total patients with distal radius fractures, and 2,608 (71.6%) met inclusion criteria. Of these, 695 (26.6%) had fracture reduction. Of the reductions, 522 (75.1%) were hematoma blocks, 151 (21.7%) procedural sedation, and 22 (3.2%) UGNB. The majority of UGNB (72.7%, n = 16), procedural sedation (64.2%, n = 97), and hematoma block reductions (51.3%, n = 268) were performed in community hospital EDs. Patient age was greatest for the hematoma block (median 67 [57, 76]), followed by no ED reduction (65 [51, 77]), UGNB (65 [51, 68]), and procedural sedation (62 [43, 72]) (*P* < .01 for the four-group comparison). Most patients (93.7%) were White and not Hispanic or Latino (94.5%). There was no difference in treatment type by race or ethnicity. Pain score reduction between arrival and last score reported in the ED was statistically greatest for the procedural sedation group (8 to 4, difference of −4 [−6, −2]), followed by UGNB (8 to 5, difference of −3 [−5, 0]), hematoma block (8 to 5, difference of −3 [−5, 0]) and no reduction (7 to 5, difference of −2 [−4, 0]), (*P* < .001). Median total milligrams of morphine equivalents was higher for UGNB (7.5 [6.8, 13.9]) and procedural sedation (7.5 [2.0, 14.0]), as compared to hematoma block (6.7 [0, 13.0]) and no ED reduction (4.0 [0.0, 7.5]) (*P* < .001). Length of stay was longest for the UGNB group (314 minutes [226, 432]) compared to hematoma block (275 minutes [204, 370]), procedural sedation (258 minutes [192, 350]) (*P* = .08), and no reduction (190 [127, 290]) (*P* < .001 for the four-group comparison). Thirty-day return rates were 16.6% for procedural sedation, 15.1% for hematoma block, 12.3% for no reduction, and 9.1% for UGNB (*P* = .18).

**Conclusion:**

Most distal radius fracture reductions were done with a hematoma block. Ultrasound-guided nerve block was a less common than procedural sedation, and done predominantly in the community EDs. Procedural sedation and UGNB were most effective to reduce pain. Triage severity scores, milligrams of morphine equivalents, and length of stay were similar between UGNB and procedural sedation.

## INTRODUCTION

Distal radius fractures are one of the most common fractures,^1^ accounting for up to 18% of all fractures in the older adult population and up to 20% of all fractures treated in the emergency department (ED).^2^–^4^ Initial treatment consists of reduction and immobilization, but there is a lack of sufficient evidence to establish the relative effectiveness of different methods of anesthesia used for reduction^5^ and to determine whether care choices vary across racial and ethnic subgroups.^6^ Pain during fracture reduction is high, even when hematoma block and pre-procedural analgesia are administered.

**Population Health Research Capsule**

What do we already know about this issue?

*Ultrasound-guided nerve block (UGNB) is a growing alternative to procedural sedation used for pain control during distal radius fracture reduction in the ED*.

What was the research question?

*Our goal was to summarize the management, pain scores, and opioid use for distal radius fracture reductions in the ED*.

What was the major finding of the study?

*Most fracture reductions were done with hematoma blocks; however, UGNB and procedural sedation lowered pain levels by 3–4 points on a 0–10 scale (P < .001)*.

How does this improve population health?

*These real-world data for management of isolated distal radius fractures may encourage greater use of ultrasound-guided nerve blocks*.

Procedural sedation is an alternative for management of distal radius fractures, but it demands staff and resource allocation.^5^ Sedation has the advantage of muscle relaxation but requires more time, resources, knowledge, and experience to avoid complications,^7^ including apnea and hypotension. This is especially true in the geriatric population and in patients with significant comorbidities. Moreover, sedation requires monitoring and post-procedure observation, although serious adverse events such as intubation or aspiration remain low.^8^,^9^

Ultrasound-guided nerve block (UGNB), such as supraclavicular or brachial plexus block, has advantages when compared to procedural sedation.^5^ The UGNB can be performed using interscalene, supraclavicular, infraclavicular, or axillary approaches,^5^ with some evidence to suggest that axillary nerve blocks significantly reduce pain when compared to hematoma blocks,^10^ and the UGNB has a low complication rate.^11^ In 2021, the American College of Emergency Physicians issued a statement that performing UGNB is considered a core skill in emergency medicine training.^12^

Our objective in this study was to describe the real-world use of various anesthetic management techniques used to assist with distal radius fracture reductions in community and academic EDs.

## METHODS

This was a retrospective, observational cohort study that followed Strengthening the Reporting of Observational Studies in Epidemiology (STROBE)^13^ and retrospective chart review^14^ guidelines for reporting. Following approval by our institutional review board, all ED visits for distal radius fractures between January 1, 2020–April 15, 2024 across our health system were identified, including academic hospitals (annual visit volume of 300,000) in Minnesota, Arizona, and Florida, as well as a network of 18 community EDs in the upper Midwest (Minnesota and Wisconsin; annual visit volume of 190,000). We did no manual data abstraction. All data were retrieved electronically from the electronic health record (Epic Systems Corporation, Verona, WI). Missing data were reported as “unknown” in our results. Eligible patients consisted of adults with isolated distal radius fractures. Patient encounters were categorized based on pain management modality: UGNB; procedural sedation; and hematoma block (for ED reductions); and no ED reduction using anesthesia procedure notes. Procedural sedation includes procedural, moderate, or deep sedation (with or without lidocaine administered in the ED). The hematoma block category includes hematoma block, local infiltration, or lidocaine administered in the ED (not in combination with UGNB or procedural sedation). Hematoma block was recorded within the clinician note. Cases with no reduction performed did not receive anesthesia or lidocaine for fracture management in the ED.

Patient demographics (including age, sex, race and ethnicity) and triage Emergency Severity Index (ESI) were recorded. The primary outcome was pain score (rated 0–10) reported at ED presentation and again prior to disposition. Secondary outcomes included total milligrams morphine equivalents administered during the encounter, ED waiting time and time in treatment room, disposition from the ED, ED length of stay (LOS) and 30-day returns. We compared characteristics and outcomes between patient groups using two-sided Kruskal-Wallis tests (numeric) and chi-squared or Fisher exact tests (categorical). *P*-values < 0.05 were considered significant.

## RESULTS

We identified a total of 3,642 ED visits for the study. After excluding 865 pediatric patients, 165 adult patients with polytrauma, and four with isolated ulnar fracture, 2,608 visits were included in the analysis. Of those included, 695 patients received fracture reduction in the ED (26.6%). Of those fractures reduced in the ED, 22 patients (3.2%) received UGNB, 151 (21.7%) received procedural sedation, and 522 (75.1%) received hematoma block. Of those fractures not reduced in the ED, 27 (1.5%) patients were sent to the operating room. The majority of UGNB (72.7%, N = 16), procedural sedation (64.2%, N = 97), and hematoma block reductions (51.3%, N = 268) were performed in community EDs.

Table 1 provides a summary of patient and ED visit characteristics for these four groups. The median age for the hematoma block, no reduction, UGNB and procedural groups was 67, 65, 65, and 62 years of age, respectively (P < .01), with ≥ 72% of each group female. Most patients (93.7%, n = 2,444) were White, with 1.9% (n = 49) Black, 1.7% (n = 45) Asian, 0.8% (N=20) of other/mixed race, 0.5% (n = 14) American Indian or Alaskan native, and 0.1% (n = 3) native Hawaiian or Pacific Islander. Most (94.5%, n = 2,465) were not Hispanic or Latino. There were no differences in sex, race, or ethnicity across treatment groups. Triage ESI was different among the groups, with 13.9% of the procedural sedation group, 9.8% of the hematoma block group, 9.1% of patients in the UGNB group, and 8.5% of the no reduction group receiving high-severity ESI scores of Level 1 or 2 (*P* < .001 across all groups

**Figure 1:**
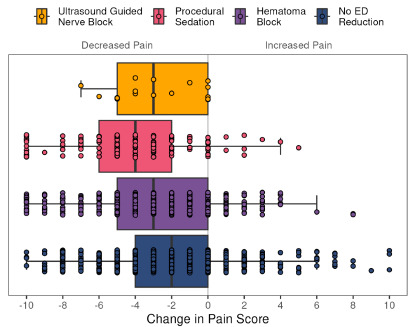
Change in reported pain score from arrival in the emergency department to last recorded pain score among patients with distal radius fractures receiving ultrasound-guided nerve block (median change −3, IQR Q1, Q3 [−5, 0]), procedural sedation (−4, [−6, −2]), hematoma block (−3, [−5, 0]), or no reduction (−2, [−4, 0]).

Initial pain scores were lowest in the no reduction group (median 7, IQR 5–9), when compared to UGNB (median 8, IQR 5–10), hematoma block (median 8, IQR 5–9) and procedural sedation (median 8, IQR 6–10) (*P* < .001), with no difference between the UGNB and procedural sedation groups (*P* = .97). The reduction in pain scores between arrival and last report in the ED were different among the groups, with a decrease of 3 points, 4 points, 3 points and 2 points for UGNB, procedural sedation, hematoma block, and no reduction, respectively (*P* < .001; Figure 1). There was no difference between the UGNB and procedural sedation groups (P = .18). Percentage of available pain scores varied, ranging from 98–100% available at presentation to 94% (procedural sedation), 85% (hematoma block), 77% (UGNB), and 69% (no reduction) available as a last score in the ED. Total milligrams of morphine equivalents administered was lowest in the no reduction group (4.0, IQR 0.0–7.5), followed by hematoma block (6.7, IQR 0.0–13.0) (P < .001), UGNB (7.5, IQR [6.8, 13.9]) and procedural sedation (7.5, IQR [2.0, 14.0]). (*P* < .001). There was no difference in milligrams of morphine equivalents administered between the UGNB and procedural sedation groups (*P* = .21).

The ED LOS was longest for the UGNB group (median 314 minutes), when compared to procedural sedation (258 minutes), hematoma block (275 minutes), and no reduction (190 minutes) (*P* < .001 for four-group comparison), although there was no difference in LOS between UGNB and procedural sedation (*P* = .08). Emergency department disposition was similar across groups, with 72.7–83.9% of all patients discharged home. Few patients required immediate admission to the operating room (0%, 0%, 0%, and 1.4%) for UGNB, procedural sedation, hematoma block, and no reduction groups, respectively. Finally, 30-day ED return rates were similar across groups, ranging from 9.1% for UGNB, 16.6% for procedural sedation, 15.1% for hematoma block, to 12.3% for no reduction.

## DISCUSSION

This study describes the real-world experience of distal radius fracture reductions in the ED across a large health system of community and academic EDs. We found that a small percentage were managed with UGNB or procedural sedation for reduction, with a very low number of patients requiring immediate surgery, and there were similar ED return rates across groups. We found no differences in procedure type by sex, race or ethnicity.

The majority of patients either did not receive fracture reduction in the ED or required hematoma block for fracture reduction. The no reduction group had the least severe triage scores on presentation, lowest milligrams of morphine equivalents needed for pain management and the shortest ED LOS, while patients with the highest levels of intervention (UGNB and procedural sedation) had similar severity of triage scores, milligrams of morphine equivalents administered, and ED LOS. Procedural sedation resulted in the largest decrease in reported pain score across the ED visit timeline, although not statistically less than for UGNB. Type of clinician doing the reduction was not uniformly available. Given that pain score reporting was highest for procedural sedation and UGNB post-procedure and least for no reduction, pain reduction scores may have been a focus for the care teams in those cases, potentially influencing results. Future studies involving prospective collection of pain score data would improve comparisons.

Within the hematoma block group, which consisted of the oldest patient population, some may not have been candidates for procedural sedation because of their underlying comorbidities. These patients may still have been candidates for UGNB to provide pain relief. The UGNB has many advantages over procedural sedation and hematoma block, including increased relaxation of targeted muscle groups, allowing for better quality reduction without the risks^10^ of procedural sedation.^15^ There is a low complication rate reported, with pneumothorax^15^ and hemi-diaphragmatic paresis the most common.^17^ The UGNB was performed most frequently in our community sites, with 73% of all UGNB procedures performed in community hospitals. This demonstrated good uptake of the procedure in sites with appropriately trained staff and available resources. While financial incentives may be a consideration for relative value unit-based hospital reimbursement systems, all our academic teaching hospitals and affiliated community sites operate on a salary compensation model.

The UGNB procedures performed in our sample population resulted in longer ED stays when compared to both procedural sedation and hematoma block groups. Longer ED stays may have been the result of longer waiting room times and times in the treatment room when compared to procedural sedation. The skills and time needed to prepare for UGNB may have also influenced the duration, along with clinical decision factors. In a previous small prospective trial of 12 patients,^3^ UGNB was shown to shorten ED LOS and decrease resource use. Results from that study may have been influenced by the unblinded study design and operator experience. Yet the time reported to initiation of block was also shorter than for procedural sedation in that study. Length of time to procedure in our study was not available.

Procedural knowledge and training remain barriers to implementation, as care models and resource use likely vary across institutions. This is true for our institution, as procedural sedation requires the presence and coordination of the physician, nursing staff, respiratory therapist, and a proceduralist performing the reduction, with an additional need for post-sedation monitoring. This can become problematic, since reductions may be delayed until the appropriate resources are available.

The past 15 years have seen a shift in approach to analgesia in EDs, with UGNB becoming more commonplace among academic centers.^18^,^19^ In fact, a recent survey of 108 academic EDs with active ultrasound fellowship programs indicated that all used UGNB in some capacity, representing a 16% increase in use over the prior five years, with 28% of fellowships performing supraclavicular UGNB.^20^ In this study, we demonstrated that, despite low overall use, most UGNBs took place in affiliated community hospital ED settings. This suggests the availability of equipment, tools, and knowledge expertise outside major academic centers. One explanation is that several residency graduates from our program staffed the community hospitals, and during their training they gained proficiency in performing nerve blocks, which they have continued to apply in their current community practice.

## LIMITATIONS

Our study is limited by the retrospective and descriptive nature of the analyses due to low numbers of UGNBs. Limitations for pain score data exist. Pain score is routinely recorded at triage with vital signs but is less consistently recorded post-procedure and prior to disposition. Outgoing narcotics prescriptions at discharge and outpatient follow-up could better inform pain outcomes data. Additionally, our study is based on medical record review, and information was limited to the medical record content. Procedure notes were not typically created for hematoma block and patients who had procedural sedation (with or without lidocaine administered in the ED) were classified as procedural sedation. Rescue anesthesia was not further delineated.

Finally, description of clinician type and skill level (attending, resident, nurse practitioner, physician assistant) for reductions was not possible, which may limit interpretability of pain score data. Academic center reductions were performed by orthopedic residents in conjunction with faculty oversight; UGNB and procedural sedation were performed by faculty directly or by residents under the close supervision by faculty. At community sites UGNB was performed almost exclusively by attending physicians. Our findings may not be reproducible in other facilities or populations with different characteristics, although external validity is improved given the multicenter design. As a cohort study, effect sizes and power calculations were not performed. Future research is necessary to evaluate the quality of reduction and longer. term outcomes.

## CONCLUSION

Across academic and affiliated community hospital EDs in our large healthcare system, hematoma block was the most common method for distal radius fracture reduction, especially in older patients. Procedural sedation and ultrasound-guided nerve blocks were performed on patients with the highest triage severity scores. These techniques achieved the greatest reduction in pain scores reported and required the greatest administration of milligrams of morphine equivalents for pain. The UGNB, although uncommon for distal radius fracture reduction in our sample, was performed mostly in the community ED setting and resulted in longer ED lengths of stay. Newer policy initiatives are encouraging training on UGNB techniques, which may increase uptake and use of this procedure.

## Figures and Tables

**Table 1:** Patient demographics, pain scores, and emergency department-visit parameters for patients with distal radius fractures who received ultrasound-guided nerve block, procedural sedation, hematoma block, or no reduction.

	Ultrasound-guided nerve block (UGNB) (n = 22)	Procedural sedation (PS) (n = 151)	Hematoma block (n = 522)	No ED reduction (n = 1,913)	*P*-value (4 groups)	*P*-value (UGNB vs PS)
Age, years					**<.01**	.87
Median (Q1, Q3)	65 (51, 68)	62 (43, 72)	67 (57, 76)	65 (51, 77)		
Mean (SD)	59.3 (18.6)	58.5 (19.5)	64.4 (18.1)	61.9 (19.7)		
Sex, n (%)					.27	.98
Female	16 (72.7%)	113 (74.8%)	405 (77.6%)	1,403 (73.3%)		
Male	6 (27.3%)	38 (25.2%)	117 (22.4%)	510 (26.7%)		
Race, n (%)					.63	.42
American Indian or Alaskan Native	0 (0.0%)	1 (0.7%)	1 (0.2%)	12 (0.6%)		
Asian	0 (0.0%)	4 (2.6%)	7 (1.3%)	34 (1.8%)		
Black	0 (0.0%)	5 (3.3%)	7 (1.3%)	37 (1.9%)		
Native Hawaiian or Pacific Islander	0 (0.0%)	0 (0.0%)	0 (0.0%)	3 (0.2%)		
White	21 (95.5%)	138 (91.4%)	500 (95.8%)	1,785 (93.3%)		
Other or mixed race	1 (4.5%)	0 (0.0%)	2 (0.4%)	17 (0.9%)		
Unknown/Did not disclose	0 (0.0%)	3 (2.0%)	5 (1.0%)	25 (1.3%)		
Ethnicity, n (%)					.77	.78
Hispanic or Latino	1 (4.5%)	8 (5.3%)	17 (3.3%)	72 (3.8%)		
Not Hispanic or Latino	21 (95.5%)	139 (92.1%)	494 (94.6%)	1,811 (94.7%)		
Unknown/Did not disclose	0 (0.0%)	4 (2.6%)	11 (2.1%)	30 (1.6%)		
Hospital type, n (%)					**.01**	.32
Academic	6 (27.3%)	54 (35.8%)	254 (48.7%)	836 (43.7%)		
Community	16 (72.7%)	97 (64.2%)	268 (51.3%)	1,077 (56.3%)		
Triage ESI, n (%)					**<.001**	.51
Level 1	0 (0.0%)	4 (2.6%)	5 (1.0%)	9 (0.5%)		
Level 2	2 (9.1%)	17 (11.3%)	46 (8.8%)	153 (8.0%)		
Level 3	18 (81.8%)	100 (66.2%)	370 (70.9%)	1,055 (55.1%)		
Level 4	2 (9.1%)	30 (19.9%)	101 (19.3%)	682 (35.7%)		
Level 5	0 (0.0%)	0 (0.0%)	0 (0.0%)	3 (0.2%)		
Unspecified acuity	0 (0.0%)	0 (0.0%)	0 (0.0%)	11 (0.6%)		
Pain Score, 0–10						
First score in the ED						
Median (Q1, Q3)	8 (5, 10)	8 (6, 10)	8 (5, 9)	7 (5, 9)	**< .001**	.97
Available data, n (%)	22 (100.0%)	148 (98.0%)	512 (98.1%)	2,546 (97.6%)		
Score 2 hours post-arrival						
Median (Q1, Q3)	7 (6, 8)	5 (2, 8)	7 (4, 9)	6 (4, 8)	**.03**	.22
Difference from arrival	−2 (−4, 0)	−2 (−5, 0)	−1 (−3, 0)	−1 (−3, 0)	**.003**	.52
Available data, n (%)	8 (36.4%)	82 (54.3%)	200 (38.3%)	736 (28.2%)		
Last score in the ED						
Median (Q1, Q3)	5 (3, 7)	4 (0, 6)	5 (2, 7)	5 (3, 7)	**< .001**	.08
Difference from arrival	−3 (−5, 0)	−4 (−6, −2)	−3 (−5, 0)	−2 (−4, 0)	**< .001**	.18
Available data, n (%)	17 (77.3%)	142 (94.0%)	444 (85.1%)	1,794 (68.8%)		
Total MME administered, Mg					**<.001**	.21
Median (Q1, Q3)	7.5 (6.8, 13.9)	7.5 (2.0, 14.0)	6.7 (0.0, 13.0)	4.0 (0.0, 7.5)		
Mean (SD)	12.0 (9.3)	9.3 (8.2)	9.1 (12.0)	5.8 (8.3)		
ED waiting time, minutes					**.013**	.29
Median (Q1, Q3)	10.8 (2.8, 16.0)	7.2 (1.8, 16.5)	8.3 (1.6, 29.6)	10.0 (2.4, 31.8)		
Mean (SD)	33.9 (64.6)	20.0 (43.9)	33.9 (62.1)	30.7 (53.0)		
ED time in treatment room, minutes					**< .001**	.25
Median (Q1, Q3)	242 (199, 355)	241 (181, 320)	240 (175, 329)	161 (104, 250)		
Mean (SD)	366 (320)	261 (116)	272 (151)	205 (204)		
ED LOS, minutes					**< .001**	.08
Median (Q1, Q3)	314 (226, 432)	258 (192, 350)	275 (204, 370)	190 (127, 290)		
Mean (SD)	400 (313)	281 (123)	306 (155)	236 (209)		
ED disposition, n (%)					.98	.34
Discharge	16 (72.7%)	124 (82.1%)	438 (83.9%)	1569 (82.0%)		
Hospital admit/observation, or specialty service	6 (27.3%)	25 (16.6%)	67 (12.8%)	268 (14.0%)		
Transfer to healthcare facility	0 (0.0%)	1 (0.7%)	11 (2.1%)	47 (2.5%)		
Send to operating room	0 (0.0%)	0 (0.0%)	0 (0.0%)	27 (1.4%)		
Unknown disposition	0 (0.0%)	0 (0.0%)	0 (0.0%)	1 (0.1%)		
30-day return to the ED, n (%)	2 (9.1%)	25 (16.6%)	79 (15.1%)	235 (12.3%)	.18	.36

ED, emergency department*; ESI*, Emergency Severity Index.

*LOS*, length of stay*; MME*, milligrams morphine equivalents.
